# Combining an in silico approach with an animal experiment to investigate the protective effect of troxerutin for treating acute lung injury

**DOI:** 10.1186/s12906-019-2515-7

**Published:** 2019-06-10

**Authors:** Ying Li, Pan Ma, Jin Fu, Jingjing Wu, Xue Wu

**Affiliations:** 1 Laboratory Teaching Center of Basic Medicine, Jinzhou Medical University, Jinzhou, 121001 Liaoning People’s Republic of China; 2 Dalian Institute of Chemical Physics, Chinese Academy of Sciences, 457 Zhongshan Rd., Dalian, People’s Republic of China

**Keywords:** Troxerutin, In silico prediction, Acute lung injury, Protective effect, Inflammatory response

## Abstract

**Background:**

Troxerutin (TRX), a naturally occurring flavonoid in various fruits, has been reported to exhibit numerous pharmacological and biological activities in vitro and in vivo. However, the molecular mechanisms underlying TRX as a treatment for disease are poorly understood.

**Methods:**

Using pharmacophore mapping and inverse docking, a set of potential TRX target proteins that have been associated with multiple forms of diseases was obtained. Bioinformatic analyses were performed using the Enrichr and STRING servers to analyse the related biological processes and protein-protein networks. Furthermore, we investigated the potential protective effect of TRX against lipopolysaccharide-induced acute lung injury (ALI) using a mouse model. Morphological changes in the lungs were assessed using haematoxylin and eosin staining. Inflammatory cytokines, tumour necrosis factor-α (TNF-α), interleukin-1β (IL-1β), IL-6 and IL-10 were investigated using ELISA. Activation of MAPK and NF-κB was detected using western blotting.

**Results:**

Our network pharmacology analysis revealed the existence of multiple TRX-related chemical-target interactions and the related biological processes. We found that pretreatment with TRX protected against histological changes and obviously regulated the inflammatory cell counts and inflammatory cytokine levels in bronchoalveolar lavage fluid. Based on bioinformatic and western blot analyses, TRX may exert a protective effect against ALI by inhibiting MAPK and NF-κB signalling.

**Conclusions:**

TRX can ameliorate pulmonary injury by inhibiting the MAPK and NF-κB signalling pathways and has a potential protective effect against ALI. This study may be helpful for understanding the mechanisms underlying TRX action and for discovering new drugs from plants for the treatment of ALI.

**Electronic supplementary material:**

The online version of this article (10.1186/s12906-019-2515-7) contains supplementary material, which is available to authorized users.

## Background

Troxerutin (TRX) is a natural derivative of flavonoid rutin, which is found in grains, fruits and vegetables. Previous studies have shown that TRX has multiple biological functions, such as antioxidative, anti-inflammatory and antiplatelet activities [1–3]. Pretreatment with TRX can induce cardioprotection by modulating the inflammatory response after myocardial ischaemia/reperfusion injury in rat models [4]. Pre-treatment with TRX can induce cardioprotection through modulating the inflammatory response after myocardial ischaemia/reperfusion injury in rat models [1]. TRX reportedly protects against liver inflammation by attenuating oxidative stress-mediated NAD^+^ depletion [5]. However, whether TRX preconditioning exerts a protective effect on lung injury remains unclear.

Recently, computational approaches in combination with experiments have been successfully applied to natural product research, including the objective elucidation of the mechanisms of action and the comprehensive prediction of effective therapeutic combinations [6–8]. Yang et al. systematically investigated *Ginkgo biloba* leaves for the treatment of cardio-cerebrovascular diseases in an animal model by incorporating pharmacokinetic pre-screening and network analysis [9]. Twelve active compounds and mechanisms of the Xipayi KuiJie’an enema for the treatment of ulcerative colitis were identified using a systems pharmacology approach [10]. An increasing number of studies have shown that the application of systems pharmacology provides guidance for exploring the therapeutic mechanism of compound and herbal medicine [11–13].

Acute lung injury (ALI) is a common and major cause of acute respiratory failure, characterized by severe inflammation of lung parenchyma [14–17]. Although some new therapeutic strategies have been developed, ALI remains a major cause of mortality [16, 18]. The major characteristics of ALI are respiratory dysfunction with destruction of the alveolar capillary membrane, subsequent infiltration of peripheral inflammatory cells, and release of several cytokines [19]. Intranasal administration of lipopolysaccharide (LPS) has been widely used to induce pulmonary inflammation in animal models of ALI [20–22]. The exposure of LPS to lung tissue directly induces an acute inflammatory response in the airspaces and lung parenchyma, characterized by oedema and increasing amounts of inflammatory cells and inflammatory cytokines, such as TNF-α, IL-1β and IL-6, in the bronchoalveolar lavage fluid (BALF).

A systems pharmacology approach was used to investigate the pharmacological mechanisms of TRX in this study. First, the potential molecular targets of TRX were predicted by the PharmMapper and idTarget servers. Second, multiple targets of TRX were analysed using various bioinformatic platforms, such as STRING and DAVID. Finally, the protective effect and underlying mechanisms of TRX on ALI were explored in a mouse model. Our results provide important insights into the efficiency of TRX for treating ALI.

## Methods

### Reagents

TRX (purity > 98%) was purchased from Nanjing DASF Bio-Technology Co., Ltd. (Nanjing, China). Lipopolysaccharide (LPS, *Escherichia coli* O111: B4) was provided by Sigma-Aldrich Chemical Company (St. Louis, MO, USA). The dexamethasone (Dex) sodium phosphate injection was purchased from Changle Pharmaceutical Co. (Xinxiang, Henan, China). Interleukin (IL)-1β, IL-6, IL-10, and TNF-α levels were quantified by ELISA (Biolegend, San Diego, CA, USA). The ERK, p-ERK, JNK, p-JNK, P38, p-P38 and β-actin primary antibodies were purchased from AB Clonal Biotech Co., Ltd. (College Park, MD, USA). The NF-κB p-p65 and p65 primary antibody was purchased from Cell Signaling Technology Inc. (MA, USA). Horseradish peroxidase (HRP)-labelled anti-mouse and anti-rabbit IgG (H + L) secondary antibodies were obtained from Beijing Solarbio Science & Technology Co., Ltd. (Beijing, China).

### Prediction of putative targets

The protein targets of TRX were predicted using the PharmMapper and idTarget servers [23, 24]. The optimized structure of TRX in mol2 format was submitted to PharmMapper and idTarget for prediction of proteins with three dimensional structures in the Protein databank. The lists of predicted targets that were ranked in the top 1–5% by the abovementioned servers were further annotated to screen out the putative target list pertaining to anti-inflammation activity.

### Analysis using the database for annotation, visualization and integrated discovery

Following the computational prediction of TRX, Enrichr and ClueGO APP in Cytoscape were employed to provide a biological functional interpretation of its predicted potential targets [25, 26]. To elucidate the relevant functions linked to the predicted gene list, the target genes were evaluated by Gene Ontology (GO) using Enrich. For each GO term, the *p*-value of function clustering and the p-value following multiple detection corrections, such as the Benjamini and false discovery rate (FDR) corrections, were calculated. Enrichment scores and Fisher’s exact test *P*-values were then calculated to identify which functional gene groups were significantly enriched.

### Protein-protein interaction networks

The protein IDs of these putative protein targets predicted using the PharmMapper and idTarget servers were individually submitted to Search Tool for the Retrieval of Interacting Genes/Proteins v10 (STRING 10) to obtain biological functional interpretations of their targets in *Homo sapiens* [27]. Pathway enrichment analysis was performed for the Kyoto Encyclopedia of Genes and Genomes (KEGG) enrichment by STRING, and the significantly enriched pathways were identified based on a value of *P* < 0.05.

### Animals

Male BALB/c mice were purchased and inbred in a specific pathogen-free (SPF) facility at Jinzhou Medical University. Mice (18–22 g, 8 weeks old) were housed at room temperature (24 ± 1 °C) in a 12 light/dark cycle. All experimental procedures were performed in accordance with the National Institutes of Health Guide for the Care and Use of Laboratory Animals. The protocols were also approved by the Animal Care & Welfare Committee of Jinzhou Medical University (approval ID: SYXK-liaoning-2017-0003). No mice died, and no apparent signs of exhaustion were observed during the experimental period.

### Experimental protocols

After adjusting to the environment, the mice were randomly divided into the following 4 groups, with 18 mice in each group: control, LPS, TRX (150 mg/kg) + LPS, and Dex (5 mg/kg) + LPS. In the group receiving TXR, the mice were treated with 150 mg/kg TRX in distilled water containing 0.1% Tween 80 via oral gavage once per day for 7 days consecutively. In the control and LPS groups, the mice were treated with sterile distilled water containing the TRX solvent via oral gavage daily for one consecutive week. One hour before LPS instillation, Dex (5 mg/kg) dissolved in PBS was administered intraperitoneally as a positive control. Mice from the LPS, TRX + LPS and Dex + LPS groups were anaesthetised with pentobarbital sodium (45 mg/kg, intraperitoneal injection) and intranasally administered 25 μg of LPS, which was diluted in PBS. Control mice were given PBS without LPS. Twenty-four hours after PBS or LPS treatment, the animals were euthanized by CO_2_ gas. The TRX and Dex dosages used in this study were based on previously published literature [28–33].

### Bronchoalveolar lavage fluid analysis

Bronchoalveolar lavage (BAL) was performed by three consecutive instillations of 0.5 ml of PBS (*n* = 6–8 in each group). The BAL fluid samples were centrifuged at 800×g for 10 min at 4 °C and collected for further analysis. The cell pellets were resuspended in PBS and stained using a Wright-Giemsa staining kit (Solarbio Life Sciences, Inc.). The concentrations of TNF-α, IL-1β, IL-6 and IL-10 in the supernatants of the BALF were measured using commercially available ELISA kits in accordance with the manufacturer’s protocol (Biolegend, San Diego, CA, USA). Total protein concentrations in the BALF were quantified using the BCA Protein Assay Kit.

### Histological examination of lung tissues

The mouse lung tissues (*n* = 5 per group) were fixed overnight in 10% buffered formalin, embedded in paraffin, sectioned at a thickness of 8 μm, and then stained with haematoxylin and eosin (H&E). Pathological changes in lung tissues were observed under a Nikon microscope (Nikon, Japan) using ImageJ software (NIH, Bethesda, MD, USA).

### Pulmonary oedema level

The lung tissues were separated from the mice, and their weights were recorded. Subsequently, the lung tissues were subsequently incubated at 90 °C for 24 h and weighed to obtain the dry weight (*n* = 5–7 in each group). Then, the lung W/D ratio was calculated [32, 33].

### Western blot analysis

Lung samples were lysed with lysis buffer (Beyotime, Shanghai, China). Total proteins were purified by sodium dodecyl sulfate polyacrylamide gel electrophoresis and transferred to a polyvinylidene difluoride membrane. After blocking with 5% nonfat milk, proteins were incubated with specific primary antibodies at 4 °C overnight. After washing three times with TBST, the membrane was incubated for an additional 2 h with an HRP-conjugated secondary antibody at room temperature. Finally, proteins were detected by enhanced chemiluminescence (ECL, GE Healthcare, Buckinghamshire, UK).

### Statistical analysis

Data are presented as the mean ± SEM from three independent experiments. Statistics were calculated using GraphPad Prism 5 software (GraphPad Software, San Diego, CA, USA). One-way analysis of variance (ANOVA) was utilized to compare data among multiple groups (*P* < 0.05 set as a standard to indicate significance).

## Results

### Potential targets of TRX

**Fig. 1:**
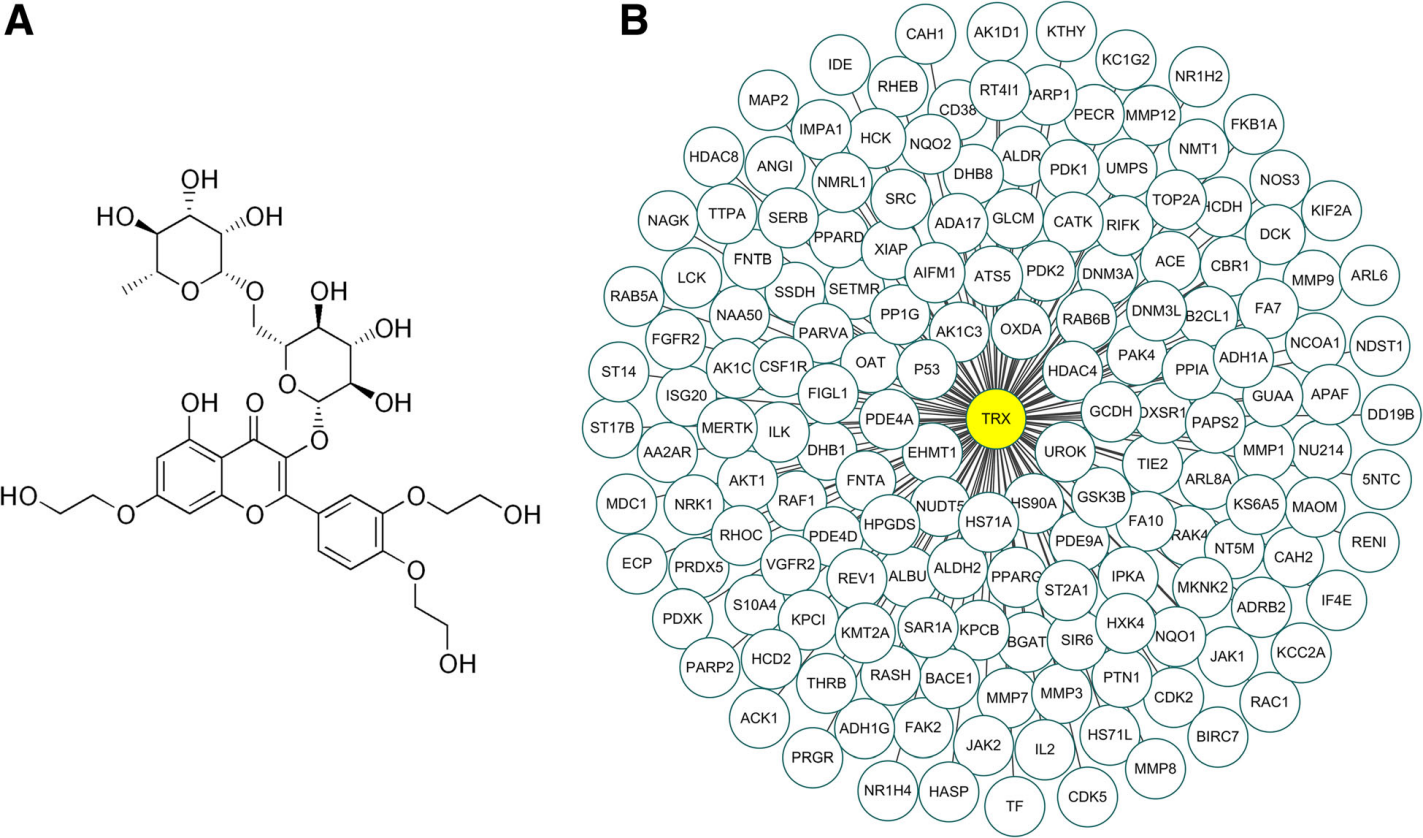
The schematic structure and the predicted target proteins of troxerutin (TRX). (**a**) The chemical structure of TRX. (**b**)162 potential targets of TRX that belong to species *Homo sapiens* had binding energies ranked in the top 100 from PharmMapper and the top 500 from idTarget

Potential targets of TRX identified by dual virtual screening procedures were analysed. Reverse pharmacology mapping was conducted using the PharmMapper server to identify potential targets of TRX with the submitted molecular structure. A list of the top 100 potential targets was obtained and further annotated to screen out the targets associated with diseases (Additional file 1: Table S1). Among the top 100 targets predicted by PharmMapper, 34 proteins belong to the species *Homo sapiens*, and among the top 500 targets predicted by idTarget, 135 proteins belong to the species *Homo sapiens* (Additional file 1: Table S1); these results are shown in Fig. 1b.

### GO analysis of predicted TRX targets

**Fig. 2:**
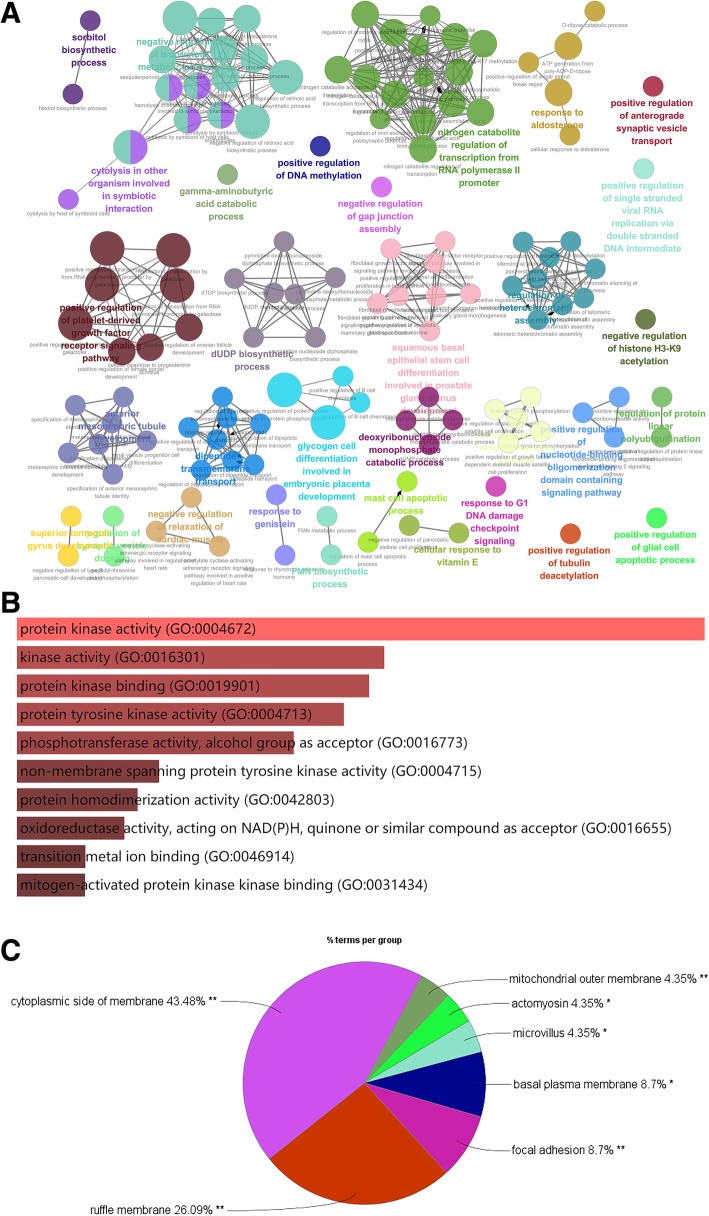
The GO analysis of predicted targets of TRX. Enrichr analysis was performed to identify the most significantly enriched GO terms. The enriched biological process terms (**a**), molecular function terms (**b**) and cellular component (**c**) were listed based on the *P*-Value

GO analysis was performed to classify the predicted targets of TRX and to identify the most significantly enriched GO terms. GO enrichment can directly reflect the distribution of target genes for each enriched GO term in the categories of biological process (BP), cellular component (CC) and molecular function (MF). As shown in Fig. 2a, some BPs were enriched, such as negative regulation of protein kinase activity by protein phosphorylation (GO:0100002), cellular response to vitamin E (GO:0071306), positive regulation of tubulin deacetylation (GO:0090044), regulation of dipeptide transmembrane transport (GO:2001148), positive regulation of tubulin deacetylation (GO:0090044), and negative regulation of relaxation of cardiac muscle (GO:1901898).

In addition, the top ten MF terms, shown in Fig. 2b, were as follows: protein kinase activity (GO:0004672), kinase activity (GO:0016301), protein kinase binding (GO:0019901), protein tyrosine kinase activity (GO:0004713), phosphotransferase activity, alcohol group as acceptor (GO:0016773) and nonmembrane spanning protein tyrosine kinase activity (GO:0004715). The main subcategories of the CC group were cytoplasmic side of membrane (GO:0098562), ruffle membrane (GO:0032587), focal adhesion (GO:0005925) and basal plasma membrane (GO:0009925).

### Protein-protein networks and pathway analysis of the predicted TRX targets

**Fig. 3:**
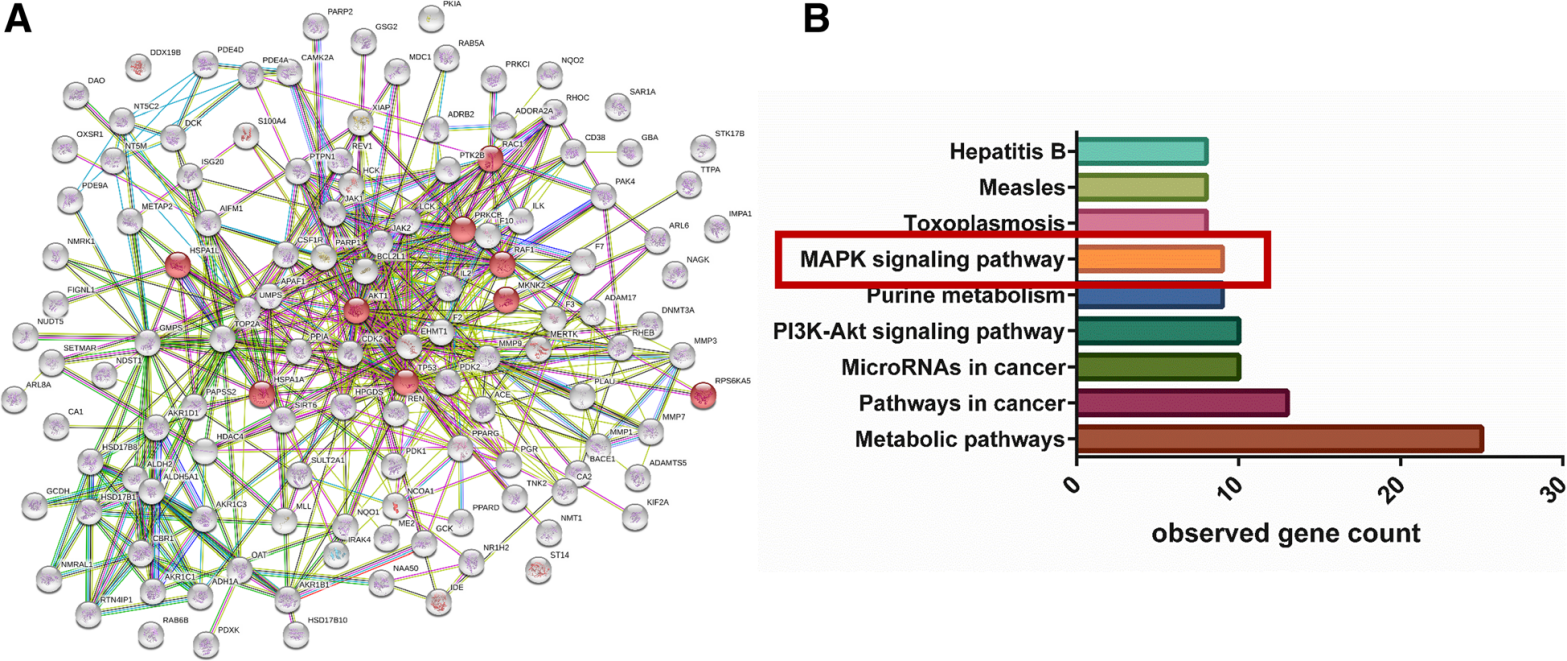
Network pharmacology analysis through the protein interaction of predicted protein targets (**a**) and signaling pathways (**b**) of representative ingredients of TRX. The network nodes were predicted proteins and the edges represented the functional associations. The red nodes were proteins included in the MAPK signaling. Pathways with a *p* value ≤0.05 after FDR correction were considered significant

To identify functional connections between the predicted proteins, protein-protein interactions were predicted using STRING-db. The human proteins and the functional interactions between them are shown in Fig. 3a. The coloured lines between the proteins indicate various types of evidence for interactions.

Next, pathway enrichment analysis was conducted. The STRING server identified 74 KEGG pathways that were significantly enriched in the target list derived from PharmMapper and idTarget (Addtional file 2: Table S2). The signalling pathways that participate in airway inflammation, including the MAPK signalling pathway (KEGG ID: 04010), VEGF signalling pathway (KEGG ID: 04370), and T cell receptor signalling pathway (KEGG ID: 04660), were found in the KEGG enrichment analysis of putative targets. In particular, MAPK signalling was ranked in the top nine signalling pathways (Fig. 3b), and nine predicted targets in the MAPK pathway may interact with TRX; these targets are labelled as red nodes in the network (Fig. 3a).

Following the bioinformatic prediction of the molecular TRX interactome, we examined the effect of TRX on ALI by assessing the inflammatory response in vivo.

### TRX modulates the pathophysiological features of LPS-induced ALI

**Fig. 4:**
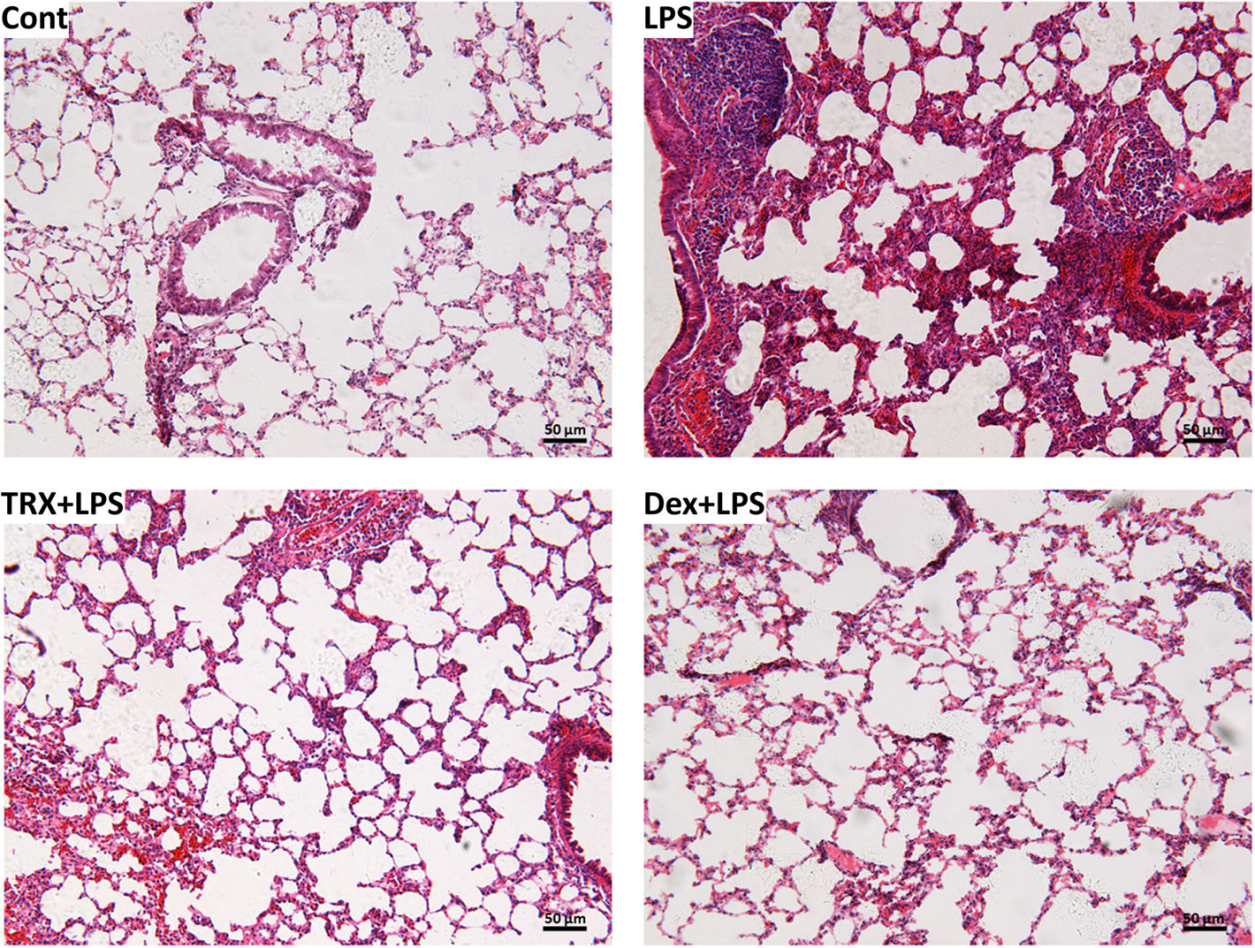
The effect of TRX on the microscopic appearances of lung from LPS-induced ALI mouse; Cont: a control group, LPS: LPS group, TRX + LPS: LPS + TRX (150 mg/kg) group, Dex + LPS: LPS + Dex (5 mg/kg) group. Histopathological sections were stained by H.E. Original magnification × 200. (*n* = 5 in each group)

As shown in Fig. 4, characteristic morphological changes, including alveolar wall thickening and obvious inflammatory cell infiltration, were observed in lung sections after LPS administration. These changes in the histopathological features of ALI were much less pronounced in the TRX pretreatment (150 mg/kg) and Dex (5 mg/kg) groups. Our results suggest that TRX and Dex significantly attenuated lung damage in LPS-induced ALI mice.

### TRX ameliorates the LPS-induced increases in lung oedema and microvascular protein permeability

**Fig. 5:**
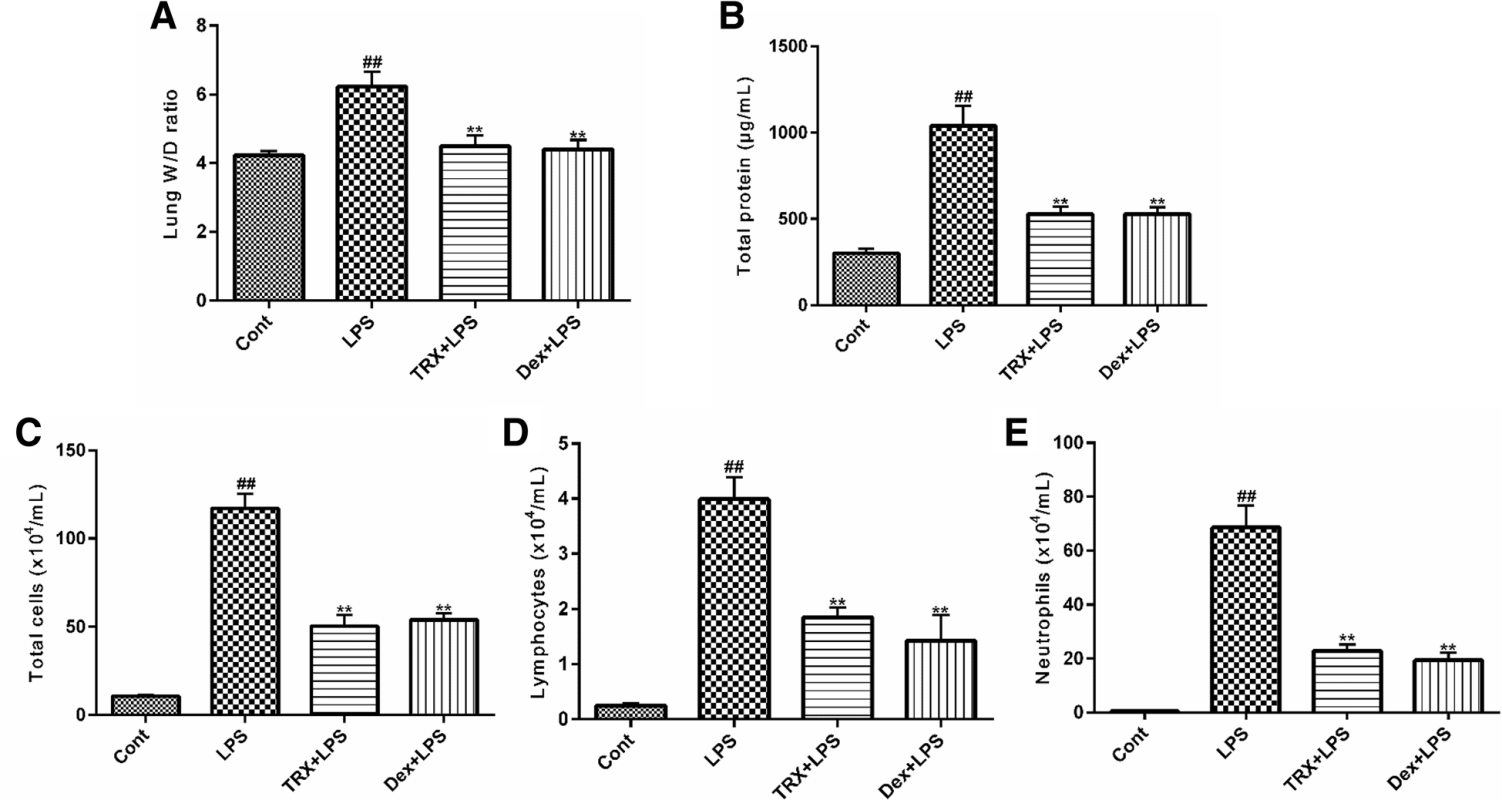
The lung W/D ratio (**a**), total protein concentration (**b**), the number of total cells (**c**), lymphocytes (**d**) and neutrophils (**e**) in the BALF was determined 24 h after LPS challenge. Data presented are the mean ± SEM (*n* = 6–8). ^##^*P* < 0.01 compared with control group, ^**^P < 0.01 compared with LPS group (one-way ANOVA)

As shown in Fig. 5a, the lung W/D ratio was higher after LPS stimulation than after the control treatment. Pretreatment with TRX (150 mg/kg) and Dex (5 mg/kg) suppressed the increase in the lung W/D ratio induced by LPS. In addition, LPS administration increased the total protein concentration in BALF compared with that achieved with control treatment. In contrast, administration of TRX or Dex prior to LPS treatment reduced the increase in the total protein concentration (Fig. 5b). These results suggest that TRX was effective in preventing lung oedema and suppressing protein leakage from the lungs of LPS-induced ALI mice.

### Effects of TRX on lung inflammatory cell accumulation in BALF

As shown in Fig. 5c-e, the accumulation of total cells, neutrophils and lymphocytes was increased after the LPS challenge compared with that achieved with control treatment. TRX (150 mg/kg) pretreatment notably reduced the total cell, neutrophil and lymphocyte counts. Our results indicate that TRX effectively suppresses LPS-induced inflammatory cell accumulation.

### Effects of TRX on the pro- and anti-inflammatory cytokine and chemokine levels in BALF

**Fig. 6:**
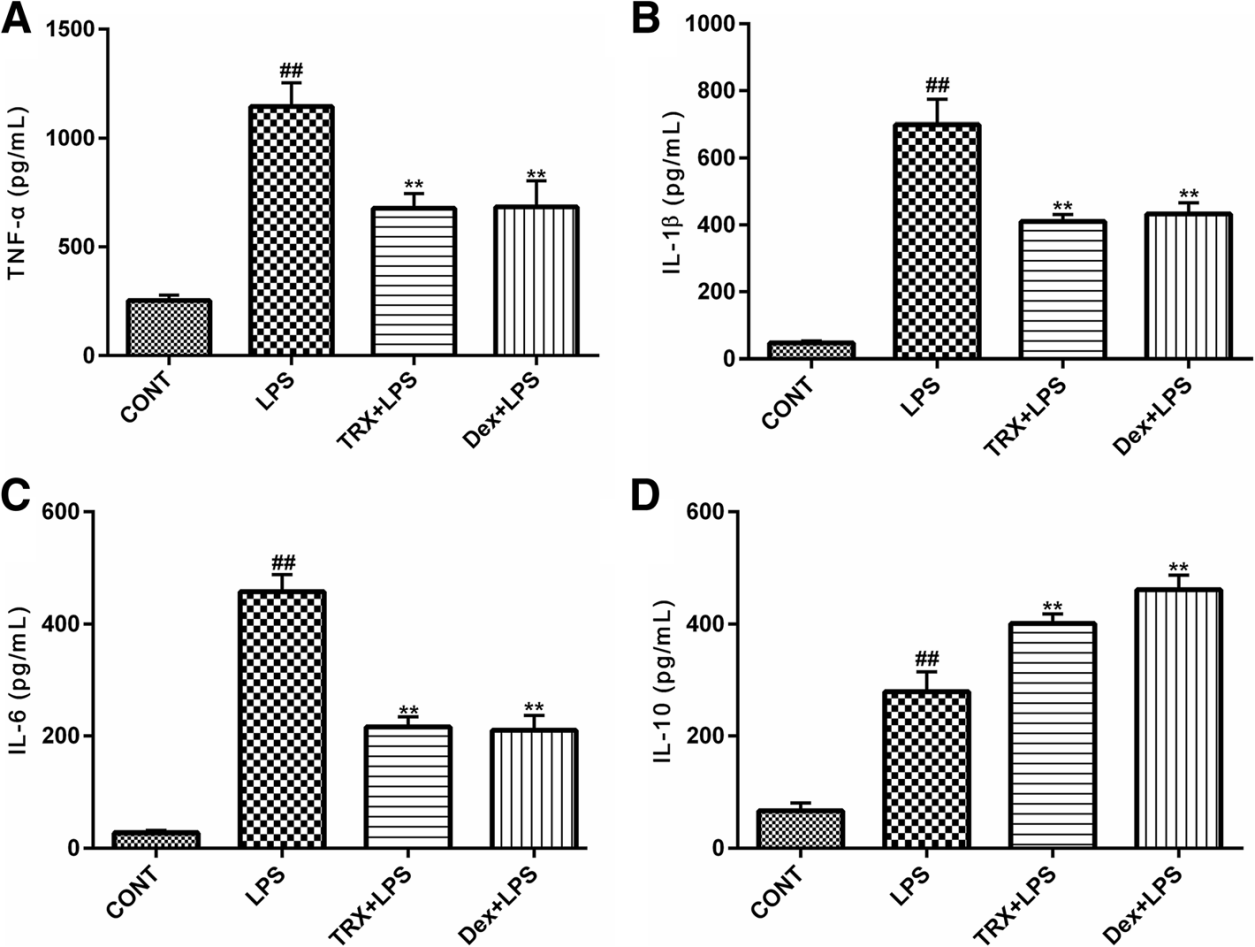
The effects of TRX on concentrations of the pro- and anti-inflammatory cytokines in BALF from mice with LPS-induced ALI. BALF were harvested to analyze the TNF-α (**a**), IL-1β(**b**), IL-6(**c**) and IL-10(**d**) using specific ELISA kits. The data presented are the mean ± SEM (n = 6–8). ^#^*P* < 0.05, ^##^*P* < 0.01 compared with control group, ^*^P < 0.05, ^**^P < 0.01 compared with LPS group (one-way ANOVA)

To assess the effects of TRX on inflammatory cytokines in LPS-stimulated ALI mice, the levels of IL-1β, TNF-α, IL-6 and IL-10 in BALF were measured by ELISA. As shown in Fig. 6, the levels of IL-1β, TNF-α, and IL-6 were significantly increased after LPS inhalation compared with those in the control group. TRX pretreatment dramatically reduced the release of TNF-α, IL-1β and IL-6 in BALF. However, the release of IL-10, an endogenous anti-inflammatory cytokine, was enhanced in the TRX and Dex groups compared with those in the LPS group. Our results suggest that TRX suppressed the release of proinflammatory cytokines and promoted the secretion of anti-inflammatory cytokines.

### Effects of TRX on MAPK and NF-κB activation

**Fig. 7:**
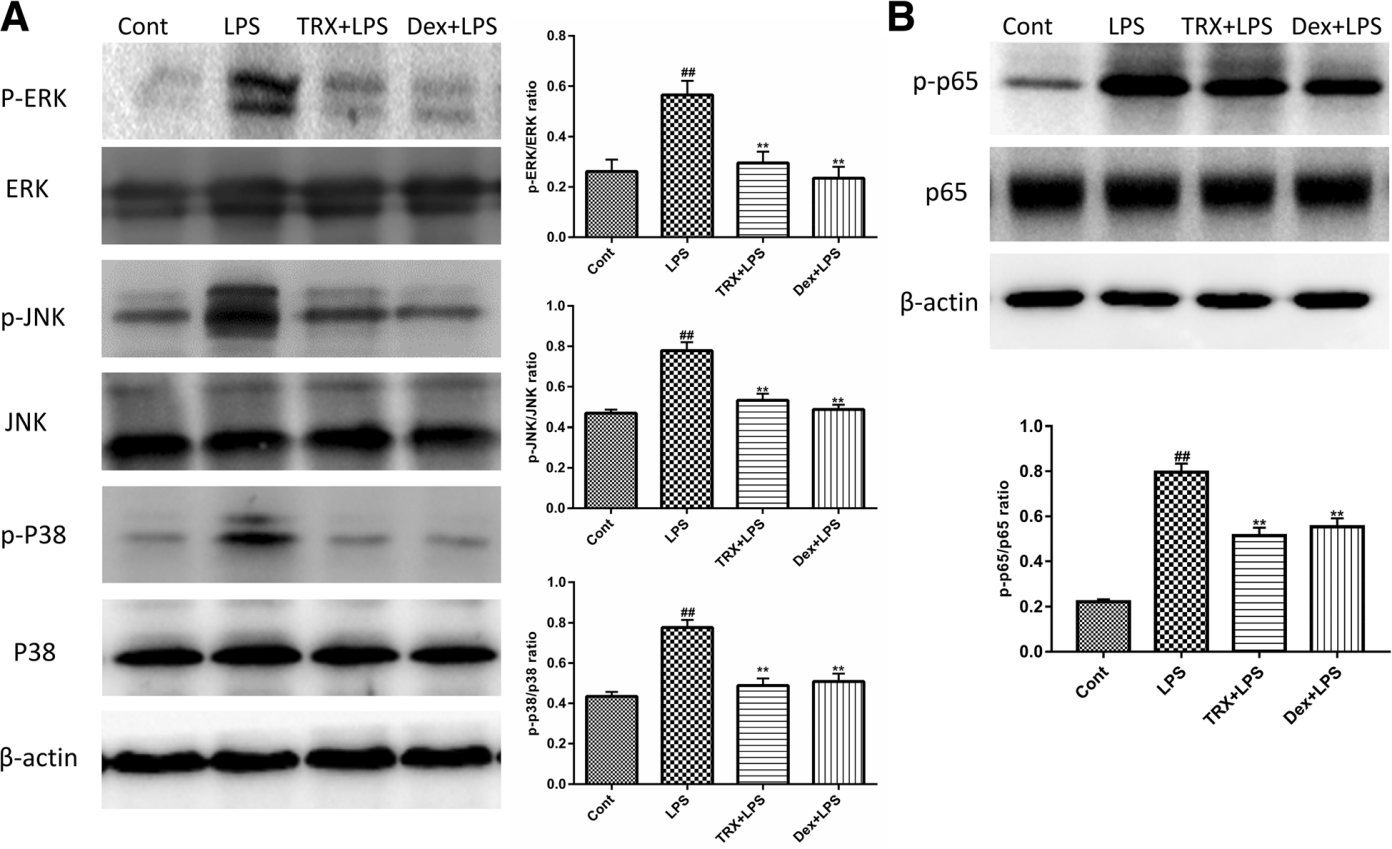
The effects of TRX on MAPK and NF-Κb pathway activation in the lung tissues from mice with LPS-induced ALI. The activation of MAPKs and NF-Κb were detected using Western blotting. Means ± SEM values of p-ERK/ERK, p-JNK/JNK, p-p38/p38 and p-p65/p65 ratios via quantitative densitometry from different groups. The assay shown is representative of three experiments with similar results. The data presented are the mean ± SEM (*n* = 5). ^#^P < 0.05, ^##^*P* < 0.01 compared with control group, ^*^P < 0.05, ^**^P < 0.01 compared with LPS group (one-way ANOVA)

As shown in Fig. 7a, the total protein levels of ERK, p38 and JNK in lung tissues remained unchanged in the LPS, TRX + LPS, and Dex + LPS groups. LPS stimulation significantly increased the phosphorylation levels of ERK, p38 and JNK. However, pretreatment with TRX or Dex markedly suppressed the phosphorylation of ERK, p38 MAPK and JNK. In addition, the phosphorylation of NF-κB p65 was markedly increased in the LPS group compared with that in the control group. However, the phosphorylated p65 proteins were reduced in the TRX and Dex groups compared with those in the LPS group (as shown in Fig. 7b). These results suggest that the beneficial effects of TRX may be partly mediated by the MAPK and NF-κB pathways.

## Discussion

In silico methodologies have become a valuable technique in drug discovery, and their impact on drug development is likely to expand [34]. Recently, a novel reverse pharmacology protocol involving a structure-based pharmacophore and inverse docking was proven to be efficient as a rapid and inexpensive method for identifying molecular targets [6, 7]. The PharmMapper online tool is a web server for potential drug target identification via reverse pharmacophore matching of the query compound against an in-house pharmacophore model database [23]. The inverse docking approach, in which ligands are docked against the protein targets in databases and the targets are scored according to the docking evaluation, has been successfully explored for target prediction [7, 35–37]. The idTarget server conducts a computer-automated search of potential targets and identifies the drug repositioning potential via inverse docking [24]. In the present study, we obtained and analysed the potential disease-related targets of TRX predicted by PharmMapper and idTarget. Our results show that TRX can modulate several signaling pathways that have important roles in the regulation of various BPs. Consistent with the bioinformatic data obtained in the present study, some previous studies regarding the mechanisms underlying TRX-regulated pharmacological effects have confirmed several TRX targets and TRX-related signalling pathways [38–40]. We found that both SRC and Akt were predicted to be possible direct protein targets of TRX according to the PharmMapper and idTarget servers, and the insulin signaling pathway (KEGG ID: 4910) was also enriched in the pathway analysis performed using STRING. Consistent with our results, Sampath reported that TRX may play a significant role in the management of type 2 diabetes mellitus by improving insulin signaling molecules, including the SRC, Akt and GLUT4 proteins [41]. In addition, TRX was reported to have anti-cancer effects, and the pathways in cancer (KEGG ID: 04064) and the NF-κB pathway (KEGG ID: 04064) were predicted to be modulated by TRX in our study [42]. A recent study reported that TRX potentiates 5-fluorouracil treatment in human gastric cancer by suppressing the STAT3/NF-κB and Bcl-2 signaling pathways [2]. A recent report indicated that TRX can exert neuroprotective effects in a 6-hydroxydopamine lesion rat model of Parkinson’s disease through the PI3K/ERβ signaling pathway (KEGG ID: 04151) [43], which was also enriched in the computational screen in our study. These data provide preliminary evidence for the validation of predicted molecular targets of TRX using a bioinformatics approach.

Several studies have noted that TRX is a potent anti-inflammatory agent, and TRX has been shown to modulate the in vivo and in vitro inflammatory responses in various disease models [44–46]. The anti-inflammatory effect of TRX has been demonstrated in D-gal-induced kidney injury through inhibition of the NF-κB mediated inflammatory response [47]. Another study reported that TRX protects against BDE-47-induced liver inflammation by attenuating oxidative stress-mediated NAD^+^ depletion [5]. In the present study, we investigated the protective effect of TRX against lung injury via bioinformatic analysis and an animal experiment. To this end, we induced ALI in mice via the intranasal administration of LPS, inducing microvascular injury, diffuse alveolar damage and neutrophil infiltration, pathological features similar to those of ALI observed in humans [16]. TRX decreased the lung W/D ratio and total protein concentration, indicating that it could effectively reduce pulmonary oedema in LPS-induced ALI mice. Activated neutrophils and alveolar macrophages induce extensive lung inflammation, resulting in destruction of the basement membrane and altered permeability of the alveolar-capillary membrane [16, 20]. In the present study, TRX suppressed the recruitment of inflammatory cells in the BALF of ALI mice. In addition, these results were supported by the histological analysis of the lungs, which revealed that the histological injuries were notably attenuated in mice administered TRX, suggesting that TRX might exert a significantly beneficial effect on LPS-induced lung injury.

Considerable evidence has emerged to indicate that restoring the pro- and anti-inflammatory balance is a potential strategy to prevent ALI [48–50]. In BALF from ALI patients, elevated concentrations of TNF-α, IL-1β, and IL-6 have been observed and correspond to an unfavourable prognosis [14]. IL-10 is capable of inhibiting the synthesis of proinflammatory cytokines, such as TNF-α and IL-1β, and lower levels of IL-10 have been observed in lungs with injury compared to those in healthy lungs [48]. In the present study, we found that TRX may protect against LPS-induced ALI by reducing the production of TNF-α, IL-1β, and IL-6 and increasing the levels of IL-10. This result is consistent with those of previous studies reporting potent regulation of inflammatory cytokine production by TRX in postconditioned rats [1].

Airway inflammation is a complex process that involves various signalling pathways, such as the MAPK, NF-κB and VEGF pathways [19, 49, 51]. In our present study, the MAPK and NF-κB signaling pathways were enriched in the set of predicted proteins. Previous research has demonstrated that MAPKs and NF-κB are important mediators involved in the release of cytokines and are associated with the pathogenesis of inflammatory processes in ALI [51–53]. Additionally, GO analysis identified “protein phosphorylation”, which is essential for MAPK activation, as a top five GO BP term. Therefore, based on this evidence, we hypothesized that the MAPK and NF-κB signalling pathways may be involved in the anti-inflammatory effect of TRX on LPS-induced ALI. In the present study, we demonstrated that the phosphorylation of ERK, JNK and p38 can be induced by LPS and that pretreatment with TRX blocks the activation of MAPKs. Consistently, previous studies reported that TRX can suppress the phosphorylation of JNK in a rat model of type 2 diabetes and block TRAF2-ASK1-JNK signalling in the mouse liver [5, 54]. NF-κB, which also serves as a major transcription factor, has been reported to play vital roles in the regulation of inflammatory cytokine production. Generally, once stimulated, the activation of NF-κB leads to the increased release of various proinflammatory cytokines, such as IL-1β, IL-6 and TNF-α. Therefore, it is important to inhibit NF-κB activation in many inflammatory diseases. In our present study, TRX administration suppressed phosphorylated p65 in LPS-stimulated ALI. However, further studies are necessary to test the effects of TRX on other pathways and their interactions.

## Conclusions

In summary, potential receptors of TRX were predicted via the PharmMapper and idTarget servers and analysed with Enrichr and STRING. TRX-related signaling pathways were obtained, and multi-scale pharmacological mechanisms of TRX were interpreted. Then, we experimentally demonstrated that TRX exerts a protective effect against LPS-induced ALI by inhibiting the MAPK and NF-κB signaling pathways. The present study provides an alternative method for the rapid identification of targets in the study of TRX or other herbal medicines and supports the potential clinical application of TRX in treating ALI.

## Additional files

Additional file 1: **Table S1**. The potential targets of TRX predicted by PharmMapper and idTarget. The top 100 best-fitted hits in PharmMapper with appropriate target annotations, as well as the aligned poses of the respective molecules are outputted. The interactome of TRX was predicated employing the idTarget server via molecular docking of a small compound across human proteome. The Docking Score fields represent the interaction strength of the molecule to the protein and the top 500 was listed. (XLSX 72 kb)

Additional file 2: **Table S2**. The results of KEGG pathway enrichment analysis. To identify functional connections between the proteins identified as predicted proteins, pathway enrichment analysis was conducted by STRING servers. The KEGG pathways were significantly enriched in the targets list derived from PharmMapper and idTarget. (XLSX 16 kb)

## Abbreviations

ALI: acute lung injury
BALF: Bronchoalveolar lavage fluid
DAVID: Database for Annotation, Visualization and Integrated Discovery
Dex: dexamethasone
ERK: extracellular regulated protein kinases
GO: Gene Ontology
H&E: haematoxylin and eosin
IL: interleukin
JNK: c-Jun N-terminal kinase
KEGG: Kyoto Encyclopedia of Genes and Genomes
LPS: lipopolysaccharide
MAPK: mitogen-activated protein kinase
PPI: Protein-protein interaction
TNF: Tumor Necrosis Factor
TRX: Troxerutin
